# Virtual Non-Contrast Reconstructions of Photon-Counting Detector CT Angiography Datasets as Substitutes for True Non-Contrast Acquisitions in Patients after EVAR—Performance of a Novel Calcium-Preserving Reconstruction Algorithm

**DOI:** 10.3390/diagnostics12030558

**Published:** 2022-02-22

**Authors:** Josua A. Decker, Stefanie Bette, Christian Scheurig-Muenkler, Bertram Jehs, Franka Risch, Piotr Woźnicki, Franziska M. Braun, Mark Haerting, Claudia Wollny, Thomas J. Kroencke, Florian Schwarz

**Affiliations:** 1 Department of Diagnostic and Interventional Radiology, University Hospital Augsburg, Stenglinstr. 2, 86156 Augsburg, Germany; josua.decker@uk-augsburg.de (J.A.D.); stefanie.bette@uk-augsburg.de (S.B.); christian.scheurig@uk-augsburg.de (C.S.-M.); bertram.jehs@uk-augsburg.de (B.J.); franka.risch@uk-augsburg.de (F.R.); piotrekwoznicki@gmail.com (P.W.); franziska.braun@uk-augsburg.de (F.M.B.); mark.haerting@uk-augsburg.de (M.H.); claudia.wollny@uk-augsburg.de (C.W.); florian.schwarz@uk-augsburg.de (F.S.); 2 Faculty of Medicine, Ludwig Maximilian University of Munich, Geschwister-Scholl-Platz 1, 80539 Munich, Germany

**Keywords:** Photon-Counting Detector CT, virtual non-contrast reconstructions, CT angiography, EVAR, radiation dose reduction

## Abstract

The purpose of this study was to evaluate virtual-non contrast reconstructions of Photon-Counting Detector (PCD) CT-angiography datasets using a novel calcium-preserving algorithm (VNC_PC_) vs. the standard algorithm (VNC_Conv_) for their potential to replace unenhanced acquisitions (TNC) in patients after endovascular aneurysm repair (EVAR). 20 EVAR patients who had undergone CTA (unenhanced and arterial phase) on a novel PCD-CT were included. VNC_Conv_- and VNC_PC_-series were derived from CTA-datasets and intraluminal signal and noise compared. Three readers evaluated image quality, contrast removal, and removal of calcifications/stent parts and assessed all VNC-series for their suitability to replace TNC-series. Image noise was higher in VNC- than in TNC-series (18.6 ± 5.3 HU, 16.7 ± 7.1 HU, and 14.9 ± 7.1 HU for VNC_Conv_-, VNC_PC_-, and TNC-series, *p* = 0.006). Subjective image quality was substantially higher in VNC_PC_- than VNC_Conv_-series (4.2 ± 0.9 vs. 2.5 ± 0.6; *p* < 0.001). Aortic contrast removal was complete in all VNC-series. Unlike in VNC_Conv_-reconstructions, only minuscule parts of stents or calcifications were erroneously subtracted in VNC_PC_-reconstructions. Readers considered 95% of VNC_PC_-series fully or mostly suited to replace TNC-series; for VNC_Conv_-reconstructions, however, only 75% were considered mostly (and none fully) suited for TNC-replacement. VNC_PC_-reconstructions of PCD-CT-angiography datasets have excellent image quality with complete contrast removal and only minimal erroneous subtractions of stent parts/calcifications. They could replace TNC-series in almost all cases.

## 1. Introduction

Endovascular aneurysm repair (EVAR) has become the primary treatment option for a wide variety of aneurysms of the descending aorta and some types of aortic dissection [1,2]. After EVAR, patients require lifelong surveillance involving regular multiphasic CT angiography (CTA) examinations. For the confident exclusion of endoleaks, a triphasic scan protocol is frequently applied, comprising a non-contrast acquisition followed by an arterial and venous phase scan [3]. These follow-up scans lead to substantial cumulative radiation exposure in this patient cohort [4,5,6].

As a dose reduction strategy for these patients, much scientific work has explored the use of dual-energy CT protocols for arterial phase acquisition. Based on dual-energy information, the iodine-containing contrast material can be quantified and subtracted, resulting in a ‘virtual non-contrast’ (VNC) series. In theory, these could be used as a substitute for a prior ‘true non-contrast’ (TNC) acquisition [1,7]; in practice, however, studies reported diverging results [8,9,10,11,12,13,14]. Therefore, initial non-contrast scans are still widely performed in this clinical context [3].

Hitherto, many of the algorithms underlying the VNC-series suffered from an imperfect differentiation of calcium vs. iodine resulting in the erroneous partial subtraction of calcium on the VNC-series. Consequently, calcifications appeared less dense on VNC- than on TNC-series, and calcium scores derived from the former had to be mathematically transformed to approximate those derived from the latter [15].

Photon-counting detector CT (PCD-CT) systems preserve the energy information of each photon generating spectral data for every scan [16,17,18]. The perfect spatiotemporal congruence of the various spectral components acquired allows for more advanced reconstruction and postprocessing applications, such as a novel multi-step calcium-preserving extension of the VNC algorithm. The routine availability of spectral data, together with these novel VNC algorithms, might obviate the need for initial non-enhanced scans in EVAR patients. This study analyzes if and to what extent this hypothesis holds true.

## 2. Materials and Methods

### 2.1. Patient Selection

The local institutional review board approved this retrospective study with a waiver for informed consent. Consecutive patients status post endovascular aneurysm repair (EVAR) who had undergone clinically indicated CT of the aorta on a novel PCD-CT between May and July 2021 were included according to the following inclusion criteria: (1) the patient was status post-EVAR with aortic stent prosthesis, (2) the patient had undergone a biphasic CT scan of the aorta (non-contrast scan and arterial phase scan), (3) CT raw data had successfully been transferred to a long-term archive.

### 2.2. CT Scan Protocol, Contrast Protocol, and Radiation Dose

All scans were acquired on a novel dual-source PCD-CT (NAEOTOM Alpha, Software Version Syngo CT VA40, Siemens Healthineers, Erlangen, Germany) in the supine position. For the non-contrast scan, the scan range was restricted to the stent-graft location, either the chest or abdomen. According to institutional standards, the CTA scan range included chest and abdomen for stent-grafts in the thoracic aorta but was restricted to the abdomen for stent grafts in the abdominal aorta.

Non-contrast scans were performed at 120 kVp tube voltage. Because of the novelty of the scanner, protocols for thoracic stent graft follow-up initially equaled those for the workup of suspected acute aortic pathologies, applying a moderately higher dose for the unenhanced scan (image quality level: 90). After a few weeks, this was reduced to 41 and affected the last two including patients with thoracic stent grafts. All patients with abdominal stents graft underwent unenhanced scanning at an image quality level of 41.

CTAs were performed with a tube voltage of 120 kVp and an image quality level of 64. Pitch was 1.5 for abdominal stent-grafts and 3.4 for thoracic stent-grafts to eliminate motion artifacts.

All scans were performed using a spectral acquisition technique (‘Quantum plus’, Siemens Healthineers) with detector-based primary thresholding of 20, 35, 65, and 70 keV.

The contrast material protocol was biphasic: 100 mL of iodinated contrast material (Ultravist 300, iopromide, Bayer, Leverkusen, Germany) were injected at 5 mL/s via an antecubital vein followed by a saline bolus of 30 mL injected at the same flow rate. Arterial phase scans were started using a bolus tracking technique; with a delay of 7 s after intraluminal enhancement in the ascending or abdominal aorta (depending on the stent graft position) reached 150 HU.

Dose Length Product (DLP) was used as an estimator for effective radiation dose and was retrieved from the automatically archived patient protocol along with the volumetric CT Dose Index (CTDI_Vol_).

### 2.3. Image Reconstruction

Axial TNC-series were reconstructed on the scanner console as 70 keV mono-energetic images using a body kernel (Br40, QIR 3, Siemens Healthineers) with a slice-thickness and increment of 5 mm and matrix size of 512. The novel PureCalcium algorithm has recently been described in detail by Emrich et al. [19]; briefly, the algorithm performs a series of routines to subtract iodine while preserving calcium. This comprises an initial detection step in which, based on the spectral properties of calcium, a ‘non-calcium mask’ is generated; after a denoising step performed by application of spectral iterative reconstruction (Q.I.R., Siemens Healthineers), this mask helps preserve full calcium contrast despite subsequent spectral iodine removal from the dataset in VNC_PC_ (PureCalcium VNC) series.

Using raw data of the CTA scan, conventional VNC-series (VNC_Conv_) were reconstructed on the scanner console, while reconstruction of VNC_PC_-series was performed on an offline workstation (RekonCT, version 15.055, Siemens Healthineers). For all VNC-reconstructions, the same axial orientation, kernel (Br40, QIR3), matrix size, and slice thickness/increment (5 mm) settings were applied as for TNC-series.

### 2.4. Image Analysis

Quantitative image analysis was performed on a dedicated workstation (Syngo.via, VB60A, Siemens Healthineers). On all TNC-, VNC_Conv_- and VNC_PC_-series, six circular regions of interest (ROIs) were positioned in the aortic lumen; two of these proximal to the stent, two within the stent lumen, and two distal to the stent. The mean and standard deviation (SD) of CT-values (in Hounsfield units, HU) were derived from all ROIs. Image noise was defined as SD of CT-values, and signal-to-noise ratio (SNR) was calculated using the following formula:

$${SNR}_{ROI}=\frac{{Mean\;CTvalues}_{ROI}}{{SD\;CTvalues}_{ROI}}$$

Mean of noise and SNR were calculated per aortic level (proximal to, within, and distal to the stent) and on a per-patient basis.

Subjective image quality analysis was performed on a clinical PACS viewing workstation (DeepUnity 1.0, Dedalus Healthcare Group, Bonn, Germany). All analyses were independently performed by three experienced radiologists (C.SM., B.J., and S.B.) with 14, 8, and 6 years of experience in cardiovascular imaging.

To evaluate overall image quality, readers were shown axial TNC-, VNC_Conv_-, and VNC_PC_-series in random order (randomized across patients) after removing all identifying labels, including the reconstruction algorithm and scan protocol used (TNC vs. VNC_Conv_ vs. VNC_PC_). Image quality was assessed on a 5-point Likert-scale (1 = very poor/non-diagnostic, 5 = excellent/highest diagnostic quality).

Subsequently, only TNC-series were unblinded and used to create the duplets (TNC|VNC_Conv_) and (TNC|VNC_PC_) for each patient. These were again separately presented in random order (randomized across patients). Using the TNC-series as a reference, readers evaluated the presence and extent of (1) erroneous calcium removal, (2) erroneous removal of stent structures, and (3) residual contrast material using 5-point Likert-scales for each variable (1 = subtraction of all calcifications/all stent parts/significant contrast material residua, 5 = no subtraction of calcifications/stent parts/no contrast material residua). Using these duplets, experts were asked if they considered the respective VNC-series a suitable substitute for the TNC-series using a 3-point Likert scale (1 = not suited, 2 = mostly suited, 3 = fully suited); this was to be based on the estimated likelihood of a diagnostic error caused by its use (1: diagnostic error possible, 2: diagnostic error conceivable but very unlikely, 3: diagnostic error inconceivable).

### 2.5. Statistical Analysis

Statistical analyses were performed using R version 4.1.0 (R Foundation for Statistical Computing, Vienna, Austria, https://www.R-project.org/, accessed on 1 December 2021). The Shapiro-Wilk test was applied to assess normality, and continuous data were compared using the t-test if normally distributed; otherwise, the Wilcoxon-Mann-Whitney test was used. Differences between groups with categorical variables were assessed using the Chi-squared test. Differences in quantitative parameters of >2 groups were assessed using the Friedman test with subsequent pairwise comparison using the Wilcoxon signed-rank test. To assess inter-reader agreement, Fleiss’ Kappa was calculated and interpreted as follows: <0.00, poor agreement; 0.00–0.20, slight agreement; 0.21–0.40, fair agreement; 0.41–0.60, moderate agreement; 0.61–0.80, substantial agreement and >0.81, excellent agreement [20]. Data are presented as mean ± SD, as median with interquartile range (IQR), or with 95% confidence interval (95% CI) as individually indicated. Differences were assumed statistically significant at *p*-values ≤ 0.05.

## 3. Results

### 3.1. Patients, Scan Parameters and DLP

Twenty consecutive patients after EVAR who had undergone biphasic CT scans of the aorta (non-contrast and arterial phase) on a novel PCD-CT were included in this study. The cohort consisted of 17 men and 3 women (age 68.5 ± 9.8; range: 52–84). Eleven patients had stent-grafts in the descending thoracic aorta and were scanned with a pitch of 3.4; nine patients had stent-grafts in the abdominal aorta and were scanned with a pitch of 1.5. Table 1 details patient demographics and scan parameters.

### 3.2. Quantitative Image Analysis

Image noise in VNC_PC_-series more closely resembled TNC-series (Δnoise_VNC-PCvsTNC_ = 1.8; *p* < 0.001) than image noise in VNC_Conv_-series (Δnoise_VNC-Conv vsTNC_ = 3.7; *p* < 0.001). SNR was lower both in VNC_PC_- (ΔSNR = 0.8; *p* < 0.001) and VNC_Conv_-series (ΔSNR = 1.4; *p* < 0.001) than in TNC-series. Quantitative image parameters are presented in detail in Table 2.

### 3.3. Subjective Image Quality Analysis

**Figure 1:**
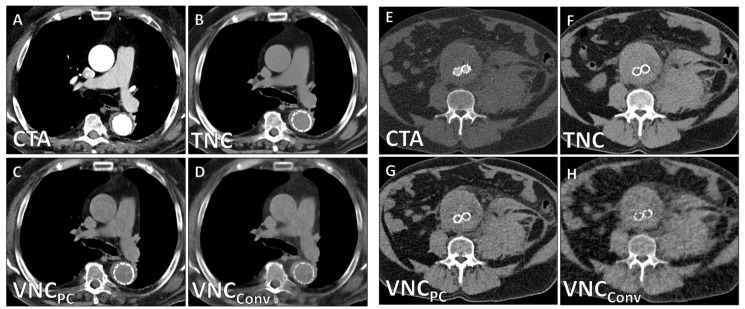
Representative PCD-CT images of a 72-year-old male patient after thoracic EVAR (**A**–**D**) and a 67-year-old male patient after abdominal EVAR (**E**–**H**).

Subjective image quality was substantially higher in VNC_PC_-series than in VNC_Conv_-series (4.2 ± 0.9 vs. 2.5 ± 0.6; *p* < 0.001). Representative images of the TNC-series and VNC_Conv_-/VNC_PC_-series are presented in Figure 1.

**Figure 2:**
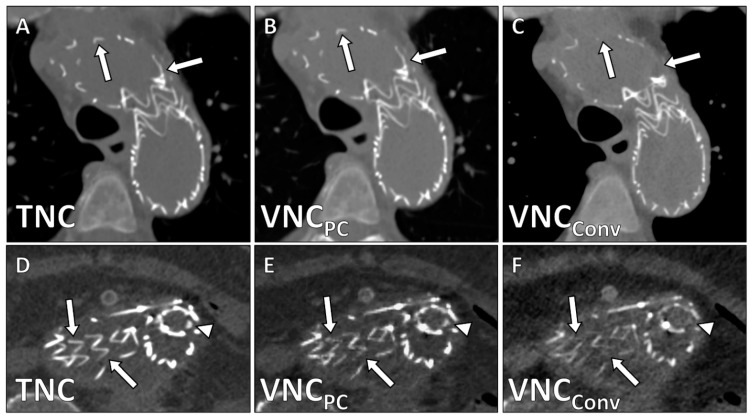
Erroneous removal of stent struts in VNC_Conv_-reconstructions demonstrated in corresponding axial images in a patient after thoracic EVAR. White arrows demonstrate stent struts (reference: TNC-series **A**,**D**) that despite lower density are well visualized in VNC_PC_-reconstructions (**B**,**E**) but are absent in VNC_Conv_-reconstructions (**C**,**F**).

**Figure 3:**
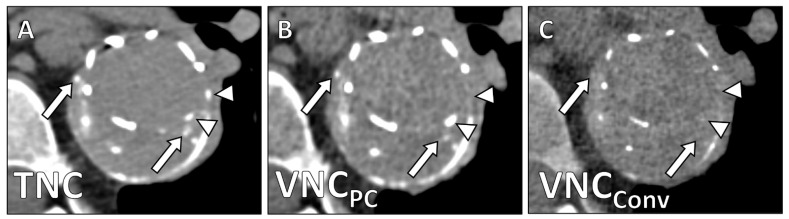
Stent after EVAR in the thoracic aorta with peripheral and central calcifications in the aneurysm sac. (**A**) TNC-series, (**B**) VNC_PC_-series, and (**C**) VNC_Conv_-series. White arrows point to calcifications that show lesser density in VNC_PC_- and full subtraction in VNC_Conv_-reconstructions. White arrowheads show missing stent struts in VNC_Conv_-series.

Unlike in VNC_Conv_-series, in VNC_PC_-series only tiny parts of stents (4.7 ± 0.7 vs. 3.8 ± 1.2; *p* = 0.003) and calcifications (4.6 ± 0.5 vs. 3.0 ± 0.6; *p* < 0.001) were erroneously subtracted (Figure 2 and Figure 3).

**Figure 4:**
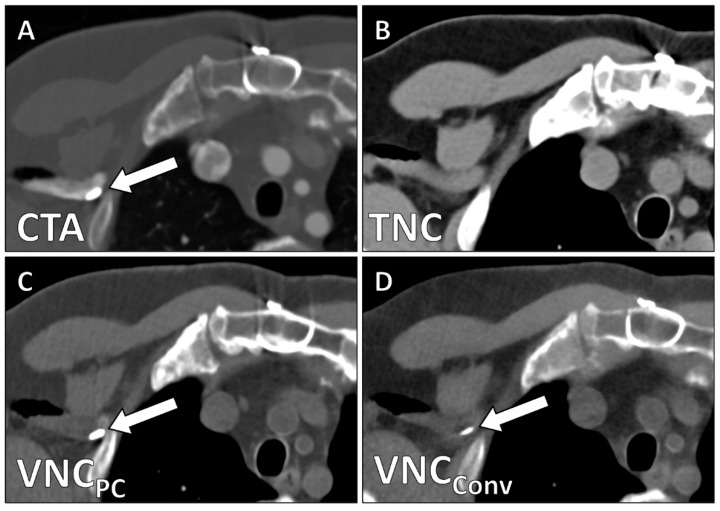
High-density contrast accumulation in the right subclavian vein (white arrow) is visible in the arterial phase scan (**A**) but not in the TNC-series (**B**). Residual non-subtracted contrast in both VNC_PC_- (**C**) and VNC_Conv_-series (**D**).

Contrast removal in the aorta was complete for all patients in both VNC_Conv_- and VNC_PC_-series. In 5 cases (25%, 5/20), there was incomplete subtraction of contrast material in the subclavian vein (Figure 4) which might be attributed to the high density of undiluted contrast material at this location.

Table 3 provides an overview of the subjective image quality parameters. There was no difference in subjective image qualities between patients scanned with a pitch of 3.4 (n = 11) and patients scanned with a pitch of 1.5 (d.n.s.).

Readers considered 95% (19/20) of VNC_PC_-series as suitable substitutes for TNC-series (75% (15/20) fully suited, 20% (4/20) mostly suited), while only 75% (15/20) of VNC_Conv_-series were deemed most suited (and none fully suited) to replace TNC-series (Χ^2^ = 11.02; *p* = 0.004).

## 4. Discussion

In this study, we evaluated VNC_Conv_- and VNC_PC_-series derived from CT angiographies of the aorta on a novel PCD-CT as potential substitutes for TNC-series in follow-up scans after EVAR. The main findings of our study are: (1) expert readers rated 95% (19/20) of VNC_PC_-series as fully or mostly suited to replace TNC-series; (2) VNC_PC_-series exhibited high image quality with complete aortic contrast removal and only minimal erroneous subtraction of stent parts or calcifications; (3) VNC_PC_-series showed lower image noise, higher SNR, and smaller CT-value differences to TNC-series than VNC_Conv_-series.

Since the introduction of DECT, many studies have explored using material differentiation algorithms to generate VNC-series from contrast-enhanced datasets as substitutes for TNC-series in various clinical scenarios [7,10,11,20,21,22,23,24] but reported diverging results. Furthermore, these approaches require access to a DECT-scanner and usually the selection of specific dual-energy acquisition protocols before image acquisition. Thus, VNC-TNC-substitution-strategies have not found wide clinical adoption. With PCD-CT systems routinely providing spectral information, one of the significant hurdles for the clinical implementation of the VNC-series has been eliminated [16,17,18,25,26,27]. The reliable availability of VNC-series would considerably lower cumulative radiation dose in many patient cohorts, particularly in patients post-EVAR who require lifelong follow-up imaging.

With endoleaks being the most common complication after EVAR, their safe exclusion is of paramount concern [28,29]. To be suitable substitutes for TNC-series, VNC-series must meet the following requirements: (1) reliable virtual contrast removal in the region of interest; (2) preservation of all calcifications, as erroneously subtracted calcifications might lead to the false diagnosis of an endoleak; (3) preservation of all stent struts to avoid the false diagnosis of stent fractures or displacement.

Both VNC_Conv_- and VNC_PC_-series showed complete contrast removal in all regions of interest. In some cases, undiluted contrast material in remote veins was still visible. However, due to the venous localization, this did not compromise image quality.

In contrast to VNC_Conv_-series, VNC_PC_-series overwhelmingly preserved calcifications. Even though calcifications and individual stent struts appeared slightly less dense and sometimes ‘sharper’ than on TNC-series in some cases, there was only a single instant (5%, 1/20) in which a solitary small calcification was erroneously removed. This remained the only case in which readers judged VNC_PC_-series as not suited to replace TNC-series; in 95% (19/20) of cases, however, readers considered VNC_PC_-series fully or mostly suited. In VNC_Conv_-reconstructions, on the other hand, smaller calcifications and parts of the stent were often entirely removed, clearly prohibiting their use as TNC-substitutes.

Although CT-value consistency between TNC- and VNC-series has been reported for DECT data, we found small HU-deviations for both VNC_PC_- and VNC_Conv_-reconstructions (D: 5.3 HU and 11.2 HU, respectively). These deviations may be related to the novelty of the scanner, data type, and algorithm and might be corrected in the future. However, for their use as TNC-substitutes, these minor CT value deviations are irrelevant. Similarly, the marginally higher image noise in VNC_Conv_- and VNC_PC_-series was associated with a slightly reduced subjective image quality but was not considered prohibitive for their use as TNC-substitutes.

Overall, VNC_Conv_-series showed significantly lower subjective and objective image quality than both TNC-series and VNC_PC_-series and exhibited relevant subtractions of stent parts or calcifications. In summary, our study provides the first evidence that based on spectral PCD-CT data, VNC_PC_-series with good to excellent diagnostic quality can routinely be derived from CTA datasets acquired with pitch values of 1.5 and 3.4; and that these are considered fully or mostly suited to substitute TNC-series in post-EVAR follow-up scans by expert readers.

These results are partially in line with but also somewhat diverge from findings of previous studies that have demonstrated very high diagnostic qualities for VNC-series [8,9,10,11,14]. One reason might be that the VNC_Conv_-reconstruction algorithm is optimized for DECT data rather than spectral data from PCD-CT. It should be noted that this study presents our initial clinical experience on a novel PCD-CT scanner. Future adjustments of the reconstruction algorithms (e.g., an adaption of VNC_Conv_-reconstructions for spectral data) will likely improve their diagnostic performance further.

This study has several limitations: First, we used raw data acquired on a novel CT scanner and a prototypic reconstruction algorithm not commercially available at the time of data acquisition. There will likely be technical adjustments to this algorithm, which might further improve the diagnostic quality of the VNC-series. Second, at the time of data acquisition, the generation of VNC_PC_-reconstructions from CT raw data required a dedicated workstation and a processing time of 20–45 min. Implementation in clinical routine will only be feasible once VNC_PC_-series can be generated on the scanner without significant delay. Third, some stent-grafts use a polymer-based sealing technique frequently resulting in prolonged contrast material retainment within the polymer [30]; in these cases, VNC series are of limited use and TNC acquisitions will likely still be mandatory for follow-up scans. Fourth, this is a retrospective single-center study with somewhat heterogeneous scan protocols. Certainly, larger studies in a prospective study setting need to confirm these findings in patients representing a broader clinical spectrum. That our results hold true regardless of whether one tube-detector system (pitch: 1.5) or both (pitch: 3.4) are used can also be viewed as a strength of our study.

## 5. Conclusions

VNC_PC_-reconstructions from PCD-CT angiography datasets have high image quality with complete aortic contrast removal and only minimal erroneous subtraction of stent struts and calcifications and are considered as fully or mostly suited to substitute TNC-series in almost all cases. This strategy should significantly reduce cumulative radiation dose in patients post-EVAR.

## Figures and Tables

**Table 1:** Patient Characteristics and Dose Parameters.

	Number of Patients in Each Group/Subgroup (n)	Value
**Age**, years	20	68.5 ± 9.8
**Gender**, male		17 (85%)
**Total DLP**, mGy·cm	20	442 ± 245
**DLP**, mGy·cm		
**Unenhanced**		
Total	20	207 ± 115
Chest	11	280 ± 107
Abdomen	9	117 ± 30
**CTA**		
Total	20	229 ± 134
Chest + Abdomen	14	315 ± 125
Abdomen only	6	124 ± 23
**CTDI_vol_**, mGy		
**Unenhanced**		
Total	20	6.0 ± 3.0
Chest	11	7.9 ± 2.8
Abdomen	9	3.6 ± 0.7
**CTA**		
Total	20	3.4 ± 1.7
Chest + Abdomen	14	4.3 ± 1.7
Abdomen only	6	2.2 ± 0.4

Data are n (percentage) or mean ± standard deviation. CTA = computed tomography angiography, CTDI_vol_ = volumetric CT dose index, DLP = dose length product.

**Table 2:** Quantitative image quality parameters compared between TNC-, VNC_PC_-, and VNC_Conv_-series.

	TNC	VNC_PC_	VNC_Conv_	Friedman X^2^	*p*	Subgroup Analysis	*p*
CT values/HU	44.4 ± 15.5	39.1 ± 15.8	33.2 ± 15.9	19.6	0.006	TNC/VNC_PC_ TNC/VNC_Conv_ VNC_PC_/VNC_Conv_	0.013 <0.001 <0.001
Noise/HU	14.9 ± 7.1	16.7 ± 7.1	18.6 ± 5.3	22.8	0.003	TNC/VNC_PC_ TNC/VNC_Conv_ VNC_PC_/VNC_Conv_	<0.001 <0.001 0.021
SNR	3.3 ± 1.6	2.5 ± 1.3	1.9 ± 0.9	25.0	<0.001	TNC/VNC_Conv_ TNC/VNC_Conv_ VNC_PC_/VNC_Conv_	<0.001 <0.001 <0.001

Data are displayed as mean ± standard deviation (SD). VNC_PC_ = PureCalcium virtual non-contrast series; VNC_Conv_ = ‘conventional’ virtual non-contrast series; SNR = signal-to-noise ratio; TNC = true non-contrast.

**Table 3:** Qualitative CT parameters compared between VNC_PC_- and VNC_Conv_-series.

	VNC_PC_	Cohen’s κ (95% CI)	VNC_Conv_	Fleiss’ κ (95% CI)	*p*
Image Quality	4.2 ± 0.9	0.68 (0.44–0.78)	2.5 ± 0.6	0.62 (0.43–0.77)	<0.001
Calcium Subtraction	4.6 ± 0.5	0.75 (0.66–0.82)	3.0 ± 0.6	0.58 (0.40–0.71)	<0.001
Stent Subtraction	4.7 ± 0.7	0.72 (0.58–0.81)	3.8 ± 1.2	0.62 (0.49–0.77)	0.003
Contrast Subtraction Aorta	5.0 ± 0.0	1.0 (1.0–1.0)	5.0 ± 0.0	1.0 (1.0–1.0)	1
Contrast Subtraction Total	4.3 ± 0.8	0.86 (0.71–0.95)	4.0 ± 1.1	0.79 (0.66–0.89)	0.091

Data are displayed as mean ± standard deviation (SD). VNC_PC_ = PureCalcium virtual non-contrast series; VNC_Conv_ = ‘conventional’ virtual non-contrast series; CI = confidence interval.

## Data Availability

The data presented in this study are available on request from the corresponding author.

## References

[1] Chaikof E.L., Dalman R.L., Eskandari M.K., Jackson B.M., Lee W.A., Mansour M.A., Mastracci T.M., Mell M., Murad M.H., Nguyen L.L. (2018). The Society for Vascular Surgery Practice Guidelines on the Care of Patients with an Abdominal Aortic Aneurysm. J. Vasc. Surg..

[2] Swerdlow N.J., Wu W.W., Schermerhorn M.L. (2019). Open and Endovascular Management of Aortic Aneurysms. Circ. Res..

[3] Smith T., Quencer K.B. (2020). Best Practice Guidelines: Imaging Surveillance after Endovascular Aneurysm Repair. Am. J. Roentgenol..

[4] de Jong P.A., Mayo J.R., Golmohammadi K., Nakano Y., Lequin M.H., Tiddens H.A.W.M., Aldrich J., Coxson H.O., Sin D.D. (2006). Estimation of Cancer Mortality Associated with Repetitive Computed Tomography Scanning. Am. J. Respir. Crit. Care Med..

[5] Brenner D.J., Hall E.J. (2007). Computed Tomography—An Increasing Source of Radiation Exposure. N. Engl. J. Med..

[6] White H.A., Macdonald S. (2010). Estimating Risk Associated with Radiation Exposure during Follow-up after Endovascular Aortic Repair (EVAR). J. Cardiovasc. Surg..

[7] Johnson T.R.C., Krauß B., Sedlmair M., Grasruck M., Bruder H., Morhard D., Fink C., Weckbach S., Lenhard M., Schmidt B. (2007). Material Differentiation by Dual Energy CT: Initial Experience. Eur. Radiol..

[8] Sommer W.H., Graser A., Becker C.R., Clevert D.A., Reiser M.F., Nikolaou K., Johnson T.R.C. (2010). Image Quality of Virtual Noncontrast Images Derived from Dual-Energy CT Angiography after Endovascular Aneurysm Repair. J. Vasc. Interv. Radiol..

[9] Buffa V., Solazzo A., D’Auria V., del Prete A., Vallone A., Luzietti M., Madau M., Grassi R., Miele V. (2014). Dual-Source Dual-Energy CT: Dose Reduction after Endovascular Abdominal Aortic Aneurysm Repair. Radiol. Med..

[10] Müller-Wille R., Borgmann T., Wohlgemuth W.A., Zeman F., Pfister K., Jung E.M., Heiss P., Schreyer A.G., Krauss B., Stroszczynski C. (2014). Dual-Energy Computed Tomography after Endovascular Aortic Aneurysm Repair: The Role of Hard Plaque Imaging for Endoleak Detection. Eur. Radiol..

[11] Toepker M., Moritz T., Krauss B., Weber M., Euller G., Mang T., Wolf F., Herold C.J., Ringl H. (2012). Virtual Non-Contrast in Second-Generation, Dual-Energy Computed Tomography: Reliability of Attenuation Values. Eur. J. Radiol..

[12] Lehti L., Söderberg M., Höglund P., Wassélius J. (2019). Comparing Arterial- and Venous-Phase Acquisition for Optimization of Virtual Noncontrast Images From Dual-Energy Computed Tomography Angiography. J. Comput. Assist. Tomogr..

[13] Lehti L., Söderberg M., Höglund P., Nyman U., Gottsäter A., Wassélius J. (2018). Reliability of Virtual Non-Contrast Computed Tomography Angiography: Comparing It with the Real Deal. Acta Radiol. Open.

[14] Martin S.S., Wichmann J.L., Weyer H., Scholtz J.E., Leithner D., Spandorfer A., Bodelle B., Jacobi V., Vogl T.J., Albrecht M.H. (2017). Endoleaks after Endovascular Aortic Aneurysm Repair: Improved Detection with Noise-Optimized Virtual Monoenergetic Dual-Energy CT. Eur. J. Radiol..

[15] Schwarz F., Nance J.W., Ruzsics B., Bastarrika G., Sterzik A., Schoepf U.J. (2012). Quantification of Coronary Artery Calcium on the Basis of Dual-Energy Coronary CT Angiography. Radiology.

[16] Flohr T., Petersilka M., Henning A., Ulzheimer S., Ferda J., Schmidt B. (2020). Photon-Counting CT Review. Phys. Med..

[17] Flohr T., Ulzheimer S., Petersilka M., Schmidt B. (2020). Basic Principles and Clinical Potential of Photon-Counting Detector CT. Chin. J. Acad. Radiol..

[18] Bette S.J., Braun F.M., Haerting M., Decker J.A., Luitjens J.H., Scheurig-Muenkler C., Kroencke T.J., Schwarz F. (2021). Visualization of Bone Details in a Novel Photon-Counting Dual-Source CT Scanner—Comparison with Energy-Integrating CT. Eur. Radiol..

[19] Emrich T., Aquino G., Schoepf U.J., Braun F., Woznicki P., Decker J.A., O’Doherty J., Brandt V., Allmendinger T., Nowak T. (2022). Coronary CTA-Based Calcium Scoring: In-Vitro and In-Vivo Validation of a Novel Virtual Non-Iodine Reconstruction Algorithm on a Clinical, First Generation Photon Counting-Detector System. Investig. Radiol..

[20] Landis J.R., Koch G.G. (1977). The Measurement of Observer Agreement for Categorical Data. Biometrics.

[21] Connolly M.J., McInnes M.D.F., El-Khodary M., McGrath T.A., Schieda N. (2017). Diagnostic Accuracy of Virtual Non-Contrast Enhanced Dual-Energy CT for Diagnosis of Adrenal Adenoma: A Systematic Review and Meta-Analysis. Eur. Radiol..

[22] Holz J.A., Alkadhi H., Laukamp K.R., Lennartz S., Heneweer C., Püsken M., Persigehl T., Maintz D., Große Hokamp N. (2020). Quantitative Accuracy of Virtual Non-Contrast Images Derived from Spectral Detector Computed Tomography: An Abdominal Phantom Study. Sci. Rep..

[23] Sauter A.P., Muenzel D., Dangelmaier J., Braren R., Pfeiffer F., Rummeny E.J., Noël P.B., Fingerle A.A. (2018). Dual-Layer Spectral Computed Tomography: Virtual Non-Contrast in Comparison to True Non-Contrast Images. Eur. J. Radiol..

[24] Si-Mohamed S., Dupuis N., Tatard-Leitman V., Rotzinger D., Boccalini S., Dion M., Vlassenbroek A., Coulon P., Yagil Y., Shapira N. (2019). Virtual versus True Non-Contrast Dual-Energy CT Imaging for the Diagnosis of Aortic Intramural Hematoma. Eur. Radiol..

[25] Decker J.A., Bette S., Lubina N., Rippel K., Braun F., Risch F., Woznicki P., Wollny C., Scheurig-Muenkler C., Kroencke T.J. (2022). Low-Dose CT of the Abdomen: Initial Experience on a Novel Photon-Counting Detector CT and Comparison with Energy-Integrating Detector CT. Eur. J. Radiol..

[26] Euler A., Higashigaito K., Mergen V., Sartoretti T., Zanini B., Schmidt B., Flohr T.G., Ulzheimer S., Eberhard M., Alkadhi H. (2022). High-Pitch Photon-Counting Detector Computed Tomography Angiography of the Aorta. Investig. Radiol..

[27] Sartoretti T., Landsmann A., Nakhostin D., Eberhard M., Roeren C., Mergen V., Higashigaito K., Raupach R., Alkadhi H., Euler A. (2022). Quantum Iterative Reconstruction for Abdominal Photon-Counting Detector CT Improves Image Quality. Radiology.

[28] Keith C.J., Passman M.A., Gaffud M.J., Novak Z., Pearce B.J., Matthews T.C., Patterson M.A., Jordan W.D. (2013). Comparison of Outcomes Following Endovascular Repair of Abdominal Aortic Aneurysms Based on Size Threshold. J. Vasc. Surg..

[29] Hobo R., Buth J., EUROSTAR collaborators (2006). Secondary Interventions Following Endovascular Abdominal Aortic Aneurysm Repair Using Current Endografts. A EUROSTAR Report. J. Vasc. Surg..

[30] de Donato G., Pasqui E., Panzano C., Brancaccio B., Grottola G., Galzerano G., Benevento D., Palasciano G. (2021). The Polymer-Based Technology in the Endovascular Treatment of Abdominal Aortic Aneurysms. Polymers.

